# Rare and massive odontogenic parakeratotic cyst treated by endoscopic sinus surgery: a case report

**DOI:** 10.1186/1752-1947-8-293

**Published:** 2014-09-05

**Authors:** Dario Marcotullio, Giannicola Iannella, Melissa Zelli, Caterina Marinelli, Giuseppe Magliulo

**Affiliations:** 1 Sense Organs Department, Sapienza University, Via Gregorio VII, no 80, Rome 00165, Italy; 2 Otolaryngology Department, University of L’Aquila, L’Aquila, Italy

**Keywords:** Endoscopic sinus surgery, Keratocystic odontogenic tumor, Maxillary sinus

## Abstract

**Introduction:**

Keratocystic odontogenic tumors are benign neoplasms of odontogenic origin with a potential for aggressive and infiltrative behavior. Many different treatments for this type of lesion have been reported. However, no common consensus has emerged to date regarding the most effective therapeutic approach. Cases of maxillary sinus giant keratocystic odontogenic tumors completely excised by enucleation or marsupialization via endoscopic sinus surgery are extremely rare, and, to the best of our knowledge, only one case has been described in the literature since 2005.

**Case presentation:**

We report a case of a 24-year-old Italian man who came to our department with maxillary sinus region swelling, pain and left nasal obstruction. A massive keratocystic odontogenic tumor involving the right maxillary sinus and causing focal erosions of the bony walls was diagnosed. The keratocystic odontogenic tumor was removed as much as possible by a transnasal approach using endoscopic sinus surgery, which produced optimal surgical and prognostic outcomes. Follow-up is reported for an 8-year period.

**Conclusion:**

Conservative management in this case demonstrated good therapeutic efficacy with a low risk of recurrence. For injuries involving the maxillary sinus, the possibility of decompression or marsupialization by endoscopic sinus surgery should always be considered because it demonstrated the potential to lead to excellent results even after 8 years of follow-up in our patient. To our knowledge, no case report has described follow-up longer than 8 years for a maxillary sinus keratocystic odontogenic tumor treated with endoscopic sinus surgery.

## Introduction

Keratocystic odontogenic tumors are benign neoplasms of odontogenic origin with a potential for aggressive and infiltrative behavior [1-3]. The odontogenic keratocyst was described for the first time by Philipsen [4] in 1956. In 2005, the World Health Organization (WHO) re-classified odontogenic neoformations, replacing the old definition of *odontogenic keratocyst* (OKC) with a new one, designated *keratocystic odontogenic tumor* (KCOT) [5,6]. The new classification is justified by the well-known features of this lesion in terms of growth, histological features and knowledge of underlying genetic mechanisms that may explain its development [2,5,7].

Many different treatments for this type of lesion, ranging from simple curettage to highly invasive *en bloc* resection, have been reported. However, no common consensus regarding the most effective therapeutic approach has emerged to date [1,2].

Although several cases of odontogenic parakeratotic cysts have been reported in the literature, only a few were related to the new WHO classification [1,3]. In addition, among these cases, maxillary sinus giant KCOTs completely excised by enucleation or marsupialization via endoscopic sinus surgery (ESS) have been extremely rare, and, to the best of our knowledge, only one case has been described in the literature since 2005 [3].

In this case report, we present our experience in treating a patient with a KCOT removed by means of an ESS approach. There was no recurrence during 8 years of follow-up.

## Case presentation

A 24-year-old Italian man came to our department complaining of a 3-month history of left maxillary sinus region swelling with left nasal obstruction and associated mild facial pain. He had a history of repeated cycles of antibiotic therapy without any symptomatic improvement.

**Figure 1:**
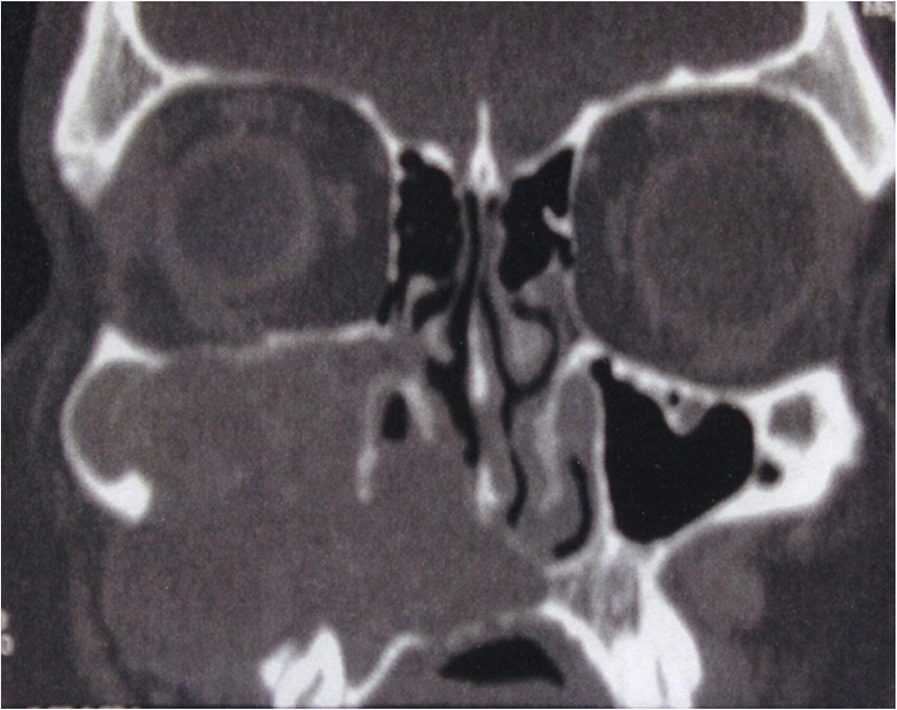
**Pre-operative computed tomography scan.** Image shows a solid neoformation occupying the right maxillary sinus, erosion of the anterior and posterior walls of the sinus and mass extension to the corresponding right dental roots and the nasal cavity, with compression and deflection of the nasal septum. There was no enhancement after contrast administration.

At the clinical examination, tumefaction was found to involve the body and ascending ramus of the maxillary sinus with intact overlying skin. No epistaxis, peri-orbital proptosis, cranial nerve palsies or paresthesias were evident. Fiber-optic rhinoscopy showed that the middle nasal meatus was partially occupied by a reddish mass that was not bleeding, together with strongly hyperemic surrounding mucosa. Erosion of the medial walls of the maxillary sinus could be seen. The right nasal cavity was apparently normal.A computed tomography (CT) scan of the paranasal sinuses showed irregularly dense solid tissue that entirely occupied the right maxillary sinus, with massive erosion of the anterior and posterior walls of the sinus. Inferiorly, the mass encased the dental roots of the corresponding right dental arch, and its extension into the nasal cavity created induced compression and deflection of the nasal septum. No enhancement after contrast administration was evident (Figure 1).

**Figure 2:**
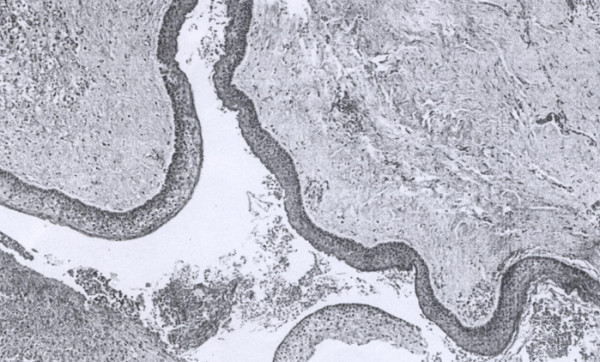
**Histological study of the patient’s keratocystic odontogenic tumor.** Image shows cell layers of squamous parakeratotic epithelium and areas of dehiscent epithelium with granulation tissue.

To remove the mass, ESS was performed. After surgical volume reduction using radiofrequency of the inferior turbinate, the maxillary ostium was enlarged and the cystic lesion was opened and decompressed. Marsupialization was performed, and the cystic wall was also removed as much as possible.The histological examination revealed the presence of a cyst wall characterized by a few cell layers of squamous parakeratotic epithelium. In some areas, the epithelium was dehiscent with granulation tissue. Lymphocytes and plasma cells could be seen (Figure 2). Such histological features were compatible with the diagnosis of KCOT.

**Figure 3:**
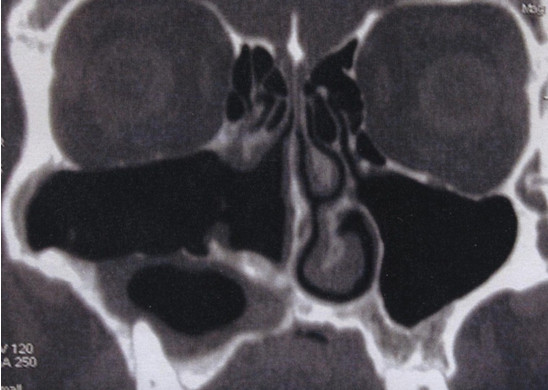
**Post-operative computed tomography scan taken at the 8-year follow-up examination.** Scan shows no keratocystic odontogenic tumor recurrence to the right maxillary sinus and scarce pathologic tissue in the alveolar recess of the right maxillary sinus.

The post-operative evaluation revealed no recurrence of the disease, with steady improvement in the patient’s clinical condition. Owing to the high tendency of KCOTs to recur, we opted for a long radiological follow-up with CT performed annually for the first 5 years and every 3 years thereafter.At the patient’s 8-year follow-up examination, a post-operative CT scan showed no KCOT recurrence in the right maxillary sinus. Scarce pathologic tissue could be seen in the alveolar recess of the right maxillary sinus (Figure 3).

## Discussion

In 2005, the new histological classification of odontogenic tumors was introduced by the WHO, which re-classified the OKC as a benign intra-osseous neoplasia, the so-called keratocystic odontogenic tumor [5,6]. Originating from the dental blade or its residue, this tumor represents 2% to 11% of all mandibular cysts [7]. Occurring at any age, these tumors primarily affect men, at an approximate ratio of 2:1 [8]. They are more often located in the mandible than in the maxilla, where they are far less frequently found [1,8]. Simiyu *et al*. [8] reported a total of 22 confirmed cases of KCOT, among which 15 (68.2%) occurred in the mandible, 6 (27.3%) occurred in the maxilla and 1 (4.5%) was in both jaws and was associated with Gorline-Goltz syndrome.

With regard to symptoms, several researchers have reported that 50% to 90% of KCOTs are symptomatic at the time of diagnosis [1]. The chief presenting symptom appears to be swelling in combination with pain, pus discharge and nasal obstruction [1,9].

KCOT is characterized by very aggressive local behavior with a high rate of recurrence, ranging from 3% to 60% [1,8-10]. It should be noted that, because of their expansive potential, KCOTs may easily reach an enormous size and occupy the entire maxilla or the nasal fossa [1,3]. Sinus infection and signs of bone erosion may also be observed. Extra-osseous occurrence of a KCOT is exceedingly rare [1,3].

KCOTs may appear as small, round or ovoid, multi-lobulated lesions with well-defined margins and a radiolucent appearance on CT scans [1]. Signs of bone erosion can often be seen [3]. A characteristic regular, parakeratinized, stratified squamous epithelium and a well-defined basal layer of columnar or cuboidal cells are among the important histological features distinguishing KCOTs from other jaw cysts [1,10]. The epithelial lining may occasionally show features of epithelial dysplasia, and the potential for malignant transformation into squamous cell carcinoma, though rare, has been reported [7,8,11].

In a series of 32 surgically treated KCOTs, Kuroyanagi *et al*. [7] observed significant expression of Ki-67 and p53 in the group with recurrences, suggesting that the evaluation of these marker proteins could help in the assessment of prognostic risks for malignant transformation and the need for eventual adjuvant treatment. KCOT recurrence rates vary enormously, from a maximum of 62% to a minimum of 0% [9-11]. Recurrence may not be a consequence of the type of surgical management, but rather a reflection of the nature of the lesion itself [2,12]. Woolgar *et al*. [11] explained that similar recurrence risk could be attributable to incomplete removal of the original cyst lining or of a new KCOT growth from small satellite cysts, or to an odontogenic epithelial residue left during surgical treatment. Moreover, the development of an unrelated KCOT in an adjacent bone region is often interpreted as a recurrence.

The appropriate treatment of KCOT lesions is still debatable [1-3]. Most surgeons advocate complete removal of the lesion with extension margins or meticulous curettage of the surrounding tissues as the best type of treatment [1,10]. Unfortunately, enucleation alone is associated with the highest recurrence rates (range, 17% to 56%), particularly when the cyst is removed in a piecemeal fashion [3,9]. To decrease the recurrence risk, various adjunctive therapies have been attempted, including peripheral ostectomy and the use of Carnoy’s solution, cryotherapy or electrocautery [1,3,10].

Recently, the surgical risk, morbidity and relatively high rate of recurrence even after the use of aggressive surgical techniques have resulted in increased use of conservative treatment [1-3,9,12]. Decompression and marsupialization are valid conservative options in the treatment of KCOTs [1-3,9]. Marsupialization was first described by Partsch [13,14] in 1882 for the treatment of cystic lesions. This technique is based on the externalization of the cyst through the creation of a surgical window in the buccal mucosa and the cystic wall. The borders are then sutured to create an open cavity that communicates with the oral cavity. This procedure relieves pressure created by the cystic fluid, allowing reduction of the cystic space and facilitating bone apposition to the cystic walls [1,13-15].

Pogrel *et al*. [15] described 10 patients with KCOTs treated by marsupialization in whom the pathology resolved completely, both clinically and radiographically.

It should be noted that if KCOTs exist in the maxilla, which is adjacent to the nasal cavity, the ESS via the transnasal approach should be considered suitable for marsupialization [3]. Ohki *et al*. [3] are the only authors to date, to the best of our knowledge, to report transnasal marsupialization using an ESS technique to remove the cystic wall and enlarge the maxillary ostium. They achieved excellent results with this procedure. We used the same surgical technique in our patient, who was KCOT recurrence-free at his 8-year follow-up examination. To our knowledge, no case report has described follow-up longer than 8 years for a maxillary sinus KCOT treated with ESS, confirming that conservative treatment may be an optimal alternative. Larger series with an adequate long-term follow-up period are required to definitively clarify the clinical validity of ESS for preventing recurrence [2].

## Conclusion

Our case report confirms the data reported in the literature to date regarding the clinical and histological features of odontogenic parakeratotic cysts. Our conservative management of our patient demonstrated good therapeutic efficacy with a low risk of recurrence. For injuries involving the maxillary sinus, the possibility of decompression or marsupialization by ESS should always be considered and has the potential to produce excellent results even after several years of follow-up.

## Consent

Written informed consent was obtained from the patient for publication of this case report and any accompanying images. A copy of the written consent is available for review by the Editor-in-Chief of this journal.

## Abbreviations

ESS: Endoscopic sinus surgery; KCOT: Keratocystic odontogenic tumor; OKC: Odontogenic keratocyst; WHO: World Health Organization.

## Competing interests

The authors declare that they have no competing interests.

## Authors’ contributions

MD acquired data. GI and MZ acquired and interpreted the data and wrote the manuscript. KM analyzed and interpreted the patient data and wrote the manuscript. GM was a major contributor in writing the manuscript, analyzed interpreted the patient data. All authors read and approved the final manuscript.
